# Fluctuating Experimental Pain Sensitivities across the Menstrual Cycle Are Contingent on Women’s Romantic Relationship Status

**DOI:** 10.1371/journal.pone.0091993

**Published:** 2014-03-19

**Authors:** Jacob M. Vigil, Chance Strenth, Tiffany Trujillo, Steven W. Gangestad

**Affiliations:** Department of Psychology, University of New Mexico, Albuquerque, New Mexico, United States of America; Knox College, United States of America

## Abstract

We explored the social-signaling hypothesis that variability in exogenous pain sensitivities across the menstrual cycle is moderated by women’s current romantic relationship status and hence the availability of a solicitous social partner for expressing pain behaviors in regular, isochronal ways. In two studies, we used the menstrual calendars of healthy women to provide a detailed approximation of the women’s probability of conception based on their current cycle-day, along with relationship status, and cold pressor pain and ischemic pain sensitivities, respectively. In the first study (*n* = 135; 18–46 yrs., *M*_age_ = 23 yrs., 50% natural cycling), we found that naturally-cycling, pair-bonded women showed a positive correlation between the probability of conception and ischemic pain intensity (*r* = .45), associations not found for single women or hormonal contraceptive-users. A second study (*n* = 107; 19–29 yrs., *M*_age_ = 20 yrs., 56% natural cycling) showed a similar association between greater conception risk and higher cold-pressor pain intensity in naturally-cycling, pair-bonded women only (*r* = .63). The findings show that variability in exogenous pain sensitivities across different fertility phases of the menstrual cycle is contingent on basic elements of women’s social environment and inversely correspond to variability in naturally occurring, perimenstrual symptoms. These findings have wide-ranging implications for: a) standardizing pain measurement protocols; b) understanding basic biopsychosocial pain-related processes; c) addressing clinical pain experiences in women; and d) understanding how pain influences, and is influenced by, social relationships.

## Introduction

It is generally assumed that gonadal sex hormones contribute to greater clinical and experimental pain experiences in women as compared to men [17], [27], [49], [56], [70]. However, research examining the associations between hormonal fluctuations across the menstrual cycle and experimental (i.e., exogenous) pain sensitivity has produced mixed findings. Some studies show that women report variability in external pain sensitivity across different phases of the menstrual cycle [26], [48], [50], [58], whereas other studies have not found these effects, leading the researchers to conclude that they do not exist [4], [42], . These discrepancies have mostly been attributed to empirical, methodological factors, such as variability in noxious stimuli induction and how menstrual phases are defined [59].

One potential conceptual confound can be derived from a *social-signaling perspective of pain* which broadly theorizes that humans experience heightened endogenous and exogenous pain sensations in the immediate presence of, and as a result of more frequent interactions with the types of social partners who are most likely to provide solicitous responses to the pain sufferer [9], [11], [68], [69], [73]. From this perspective, pain suffering behaviors (i.e., self-reports and non-verbal gestures) and pain empathizing reactions of others operate in part at the expressive-level for signaling trustworthiness cues (i.e., demonstrated vulnerability and altruism, respectively) to induce bonding with intimate (i.e., time-invested and reliable) types of relationship partners [68], [69], [73]. A basic hypothesis from this perspective is therefore that pain should be expressed in more dynamic (e.g., variable) ways for people with these types of relationships such as people who are currently in a romantic relationship. Previous research has found that people who receive higher levels of pain-related social support and solicitous behaviors from significant relationship partners tend to report greater clinical pain experiences [6], [8], [16], [18], [25], [39], [43], [51], [52], [53], [66]. Fewer studies have focused on how the individual’s naturalistic social environment may influence experimental pain reports and hence exogenous pain percepts, and the research that does exist has shown that higher levels of social support and particularly logistical support from one’s significant other is associated with greater experimental pain sensitivity, especially in women [36], [72].

In the current study, we explore the social-signaling hypothesis that variability in exogenous pain sensitivities across different fertility phases of the menstrual cycle is moderated by women’s current romantic relationship status and hence the availability of a solicitous social partner for expressing pain behaviors in regular, isochronal ways. We tested this thesis using precise, individual-level estimates of the probability of conception based on current cycle-day in separate studies using two common experimental pain protocols: an ischemic pain task (IPT) and a cold pressor task (CPT). In both experiments we used a cross-sectional sample of healthy young women who were naturally cycling or using hormonal-contraceptives and who were currently in a pair-bond or were single. We hypothesized that naturally-cycling, pair-bonded women will show greater variability in pain reports in both types of discomfort tasks than single women or women using hormonal contraceptives, as measured by the association between conception risk and pain performance. The outcome measured in the first experiment was IPT pain sensitivity. A second study was performed to examine how the probability of conception was related to CPT pain sensitivity and to improve the experimental protocol. The women in the second study came from a different geographical region, were limited to a narrower age-range, and provided more detailed information about their romantic relationship. Lastly, the discomfort task was conducted in a way that helped eliminate the potential confound of the experimenter’s presence during the CPT itself (described below).

## General Methods

### Experiment 1 Methods (Ischemic Pain Task)

#### Participants

The study protocol was approved by the University of North Florida’s Institutional Review Board and informed written consent was obtained from all participants. Undergraduate students received extra credit for their participation. Participants who self-identified contraindications to the IPT were excluded from the study, which included: any past history of illness or pathology related to peripheral vascular or neuropathic abnormalities, psychological distress/diagnoses, excessive alcohol usage in the week prior to participation, and current medication usage related to vascular or pain-related ailments. Participants endorsing any contraindication were excluded from the study. Women were drawn from a larger pool of participants and were included in the following analyses if they reported being exclusively heterosexual, if they were naturally cycling or using a hormonal contraceptive (women using any non-hormonal contraceptive techniques were excluded), and if they had not yet experienced menopause. The final sample consisted of 135 women for inclusion in the study (18–46 yrs, *M*_age_ = 22.9, *SD* = 6.0; 55% European-American, 22% African-American, 23% other ethnicity; 50% naturally cycling).

#### Ethics statement

The protocol was approved by the University of North Florida’s Institutional Review Board and two forms of written consent were obtained from all participants. The first consent form described the general research protocol, and the second described the CPT in more detail.

#### Procedures

Participants filled out questionnaires and completed the ischemic procedure. During the IPT, one male and one female researcher were present to control for audience-effects on experimental pain performance [12], [70], [71], [73]. Upon entering the ischemic task room, researchers first obtained an initial pain assessment score along a standard Visual Analogue Scale (VAS1) from 0 to 10 (from *no pain* to *worst pain imaginable*). Participants were then seated at a computer and a software program was initiated. The program provided instructions for how to indicate pain intensity ratings and pain tolerance, and it eliminated potential confounds (e.g., time latency and recording errors) that can accompany manual experimenter pain recordings. Participants were informed, by the program and the researchers, that when the task begins; to press the corresponding radio buttons to indicate their pain intensity ratings (0–10) every thirty seconds throughout the duration of the IPT (upon an audio prompt and illumination of the pain VAS) and to indicate when they desire to stop the task because they are no longer willing or able to tolerate the pain (*pain tolerance*). There was no time visible to participants on the computer screen or in the testing room in order to ensure that participants were unaware of how much time elapsed during the procedure. Participants were also informed by the researchers before they began that they could end the pain task at any time if they were no longer willing or able to continue.

#### Ischemic pain task

Once participants verbally indicated their comprehension of the task and how to use the computer interface, the IPT was initiated by first asking participants to remove any jewelry or accessories from their non-dominant arm. Participants were then asked to raise their arm above their head (so that the elbow was at ear level) for 60 seconds to ensure adequate limb de-sanguination. A sphygmomanometer (blood pressure cuff) was then placed on the participants’ forearm 5 cm above the elbow crease and manually inflated to 200 mHg over a period of 20 seconds. Participants then lowered and rested their arm at a horizontal position, and provided an initial pain assessment on the computer screen (VAS2). They were then instructed to start making continuous soft-fist movements (described as gently touching the fingertips to the palm of the hand every 3 seconds), and to continue throughout the duration of the ischemic procedure. Continuous hand flexing motions are functionally similar to handgrip exercises for quickly and reliably producing high levels of pain sensations [70], and we have found in our lab that these motions result in a more optimal (e.g., broader) range of tolerance reports. The initial pain assessment activated the program that prompted (via beep sounds and VAS illumination) participants to indicate their pain ratings every 30 seconds, over a maximum of 5 minutes and 30 seconds (VAS 3–13). Upon termination or after the maximum time had expired, the cuff was deflated over a 30-second period. Participants were unaware of this time limit and it was used to ensure the safety of the participants.

Following the ischemic procedure, participants were instructed to relax for 5 minutes to allow their pain to subside to normal levels. After a 5 minute resting period participants were asked to complete a final pain intensity rating to ensure the absence of any discomfort that resulted from the ischemic procedure for the participants’ safety. The entire discomfort task took between 6 and 15 minutes to complete.

#### Measures

The questionnaire was created by our lab as part of a larger survey that covered a wide-range of (non-pain and relationship-related) personal and psychological topics; the individual items that pertained to the current study included sex, age, ethnicity, romantic relationship status, and menstrual functioning. Current romantic relationship status was measured with a single item asking: *Are you currently in a committed/monogamous romantic relationship?* This item was dichotomously coded (single coded 0, pair-bonded coded 1). The menstrual-related information included: whether or not the participant was *currently menstruating*, usage and type of *contraceptives*, *average number of days in their typical menstrual cycle*, and *number of days since their last menstrual cycle* (from the date of assessment. Participants were provided calendars to indicate their three most recent menstrual cycles to calculate the menstrual information.

Depression has been shown to correlate with pain tolerance [1], [30], and this was measured with the *The Center for Epidemiologic Studies Depression Scale* [47]. The instrument consists of 20 items that are scored on a 4-point Likert scale (*α* = .87). Independent samples *t*-tests showed that the overall depression score was higher in natural cyclers than hormonal users *t*(133) = 2.63, *p* = .010, *d* = .46, and there was a trend for higher depression scores in single woman than pair-bonded women, *t*(133) = 1.91, *p* = .059, *d* = .33. However, the depression score was not correlated with pain tolerance (*r* = −.06), and therefore this variable was not examined any further.

#### Data analyses

Individual-level fertility level was calculated using the Wilcox findings which provide a precise estimate of the probability of conception based on a standardized 28-day cycle [77]. These estimates calculated the probability of pregnancy relative to intercourse on a given cycle day (counting from onset of previous menses). This technique provides more detailed information than a traditional split-calendar method used in previous studies [7] which instead divide women into either low- or high-fertility groups.

Because ischemic pain sensations tend to graduate quickly in some people, we computed a pain intensity score that captured the VAS ratings midway into the task. This was done by examining the pain intensity rating at 90 sec. into the pain task (VAS5) for all the subjects (*n* = 104) who endured the ischemic task for at least 90 seconds. We also included pain tolerance as an outcome measure for the entire sample (*n* = 135), due to its distinct conceptual and applied significance (e.g., for estimating pain severity vs. threshold for seeking medical attention). Multiple regressions and bivariate correlations were used to examine the relations between conception risk, romantic relationship status, and the pain intensity and pain tolerance scores.

### Experiment 2 Methods (Cold Pressor Pain Task)

#### Participants

The study protocol was approved by the University of New Mexico’s Institutional Review Board and informed written consent was obtained from all participants. Undergraduate students again received extra credit for an introductory psychology course for their participation. Participants who self-identified contraindications to the CPT were excluded from the study; these included: taking any pain medications or having a problem that would increase risk from the CPT, including illnesses related to a cardiovascular disorder (e.g., high blood pressure, heart problems, or heart rhythm concerns), history of fainting or seizures, history of frostbite, having an open cut, sore or bone fracture on the limb to be immersed in water, or a history of Reynaud’s phenomenon. The women were again drawn from a larger pool of prospective participants and were included in the following analyses if they were younger than 30 yrs, if they were naturally-cycling or using a hormonal contraceptive (women using any non-hormonal contraceptive techniques were again excluded), and if they had not yet experienced menopause. The final sample consisted of 107 women with complete data (see below) for inclusion in the study (18–29 yrs, *M*_age_ = 19.9, *SD* = 2.1; 39% European-American, 35% Latin-American, 26% other ethnicity; 56% naturally-cycling, 44% hormonal contraceptive users).

#### Ethics statement

The protocol was approved by the University of New Mexico’s Institutional Review Board and two forms of written consent were obtained from all participants. The first consent form described the general research protocol, and the second described the CPT in more detail.

#### Procedures

After informed written consent was obtained, participants completed self-report questionnaires including demographic items and the *Norbeck Social Support Questionnaire* [40], [41]. Following questionnaire completion, participants viewed an instruction video for the CPT. The video explained how to use the cold pressor apparatus and the computer software to indicate pain ratings. The surveys and instruction video took about 30 minutes to complete.

After participants viewed the instruction video, they were led into the cold pressor room, which included an intercom system, as well as the cold pressor apparatus and a laptop programmed for participants to rate their pain levels. The software recorded participants’ baseline pain and pain intensity ratings at equal intervals throughout the CPT. The cold pressor task was then carried out by the participant without an experimenter present, though these actions were monitored by an experimenter in the next room (by video and intercom) to ensure adherence to the cold pressor methods. This enabled us to collect CPT data without the physical presence of investigators, which has been shown to influence experimental pain sensitivity [24], [32], [70]. Following the CPT, individuals were debriefed.

#### Questionnaires

The demographic questionnaire asked about sex, age, ethnicity, education, and family background. The menstrual-related information included: whether or not the participant was *currently menstruating*, usage and type of *hormonal contraceptives*, *average number of days in their typical menstrual cycle*, and *number of days since their last menstrual cycle* (from the date of assessment). Participants were again provided a calendar to calculate their responses.

The *Norbeck Social Support Questionnaire* measures the quantity and quality of individuals’ social networks [40], [41]. It asks participants to list the names of up to 24 significant persons who provide personal support. For each person listed, the participant indicates the kind of relationship (spouse or partner, family member or relatives, friend, work or school associate, neighbor, etc.), which enabled us to filter participants who included a significant other among their list of intimate social network partners. Forty-three percent of the women reported being in a pair-bond with a significant other of some kind (partner, husband, boyfriend). Of the women in a pair-bond, 18% reported knowing their significant other for less than 1 year, 20% between 1 and 2 years, 31% between 2 and 5 years, and 31% for more than 5 years.

#### Cold pressor task

For the CPT, participants were randomly assigned to one of two experimental conditions: high pain (extremely cold water) and low pain (more tepid water). Participants were seated in a chair between the pressor apparatus (left side) and the laptop computer (right side) in a small room (2.0 m×2.5 m). The apparatus consisted of a small, insulated ice cooler box (5.5′′×11′′×8′′) that was fitted with a water circulator and filled with cold water that was set to induce either low or high levels of thermal discomfort. In the low pain condition, the ice-water was set to 16°C (noticeably below room temperature, but only slightly distressing), and in the high pain condition, the water was set to 5°C (quite cold, and increasingly painful with time; this produces a range of pain tolerance levels with minimal ceiling effects [5]). The analyses only included participants (*n* = 107) who had cold water temperatures within 1°C of the target temperatures, because small differences in water temperature (e.g., 2°C) can have significant effects on pain sensitivity measures [37]. Similarly, a circulator was used to prevent the water from warming around the participant’s hand [74].

The pain assessment program (on the laptop) displayed an initial screen with the CPT instructions. The researcher verbally reiterated the instructions by describing that when participants choose to begin the task (and initiate the pain assessment program), participants were instructed to first indicate their baseline (pre-manipulation) pain severity along a standard visual analog scale (VAS, 0–10 from *no pain* to *worst pain imaginable*; this baseline measure was denoted VAS1), while simultaneously submerging their left hand into the cold water to a marked line on the wrist (1′′ above the wrist joint). Participants were instructed to indicate their felt pain intensity electronically (via a clickable icon) upon an audio prompt and illumination of a pain VAS that was programmed to take place every 30 s (though the participant was not aware of this timing) throughout the duration of the CPT (VAS2–VAS11). Finally, participants were instructed to lift their hand out of the cold pressor apparatus when they decided that they could not stand the cold anymore.

Once the participants verbally indicated their understanding of the instructions, they were fitted with a finger pulsometer to monitor their heart rate during the CPT; this was done to ensure the safety of the participants. Lastly, the researcher reminded the participant that they would be recorded, and that they could begin the task whenever they desired. The researcher then left the cold pressor room and closed the door behind herself/himself. The procedure was observed on a video monitor from the next room, and the researcher returned to the experimental room to debrief the participant once they retracted their hand from the water or after the maximum duration of 5 minutes had occurred. Following debriefing, participants were asked to rest for five minutes to ensure they no longer felt any physical discomfort from involvement in the study and that their heart rate had returned to normal.

#### Data analyses

Individuals’ fertility level was again calculated using the Wilcox findings [77]. Likewise, because CPT pain sensations tend to graduate quickly in some people, while other people hit a ceiling effect (e.g., numbing) two-thirds of the way into the task (rendering measures of CPT pain tolerance somewhat challenging), we computed a pain intensity score that captured the VAS ratings midway into the task. This was done by averaging the pain intensity rating between 60 sec. and 120 sec. into the pain task (VAS3–VAS5) for all the subjects (*n* = 107) who endured the CPT for at least two minutes (56% of participants were in the non-painful condition, 44% were in painful condition). Multiple regressions and partial correlations were used to examine the relations between conception risk, relationship status, and pain intensity; mean water temperature during the CPT ([pre-task temp+post-task temp]/2) was entered as a covariate along with length of romantic relationship (for analyses that included only pair-bonded women).

## General Results

### Experiment 1 Results (Ischemic Pain Task)

Regressions run separately for the naturally cycling women and hormonal contraceptive users using the Wilcox estimate, Relationship Status (dichotomously coded), and the Wilcox by Relationship Status interaction term as predictor variables for the IPT pain intensity ratings revealed a trend for a significant (Wilcox x Relationship) interaction term for the naturally-cycling women (*β* = .47, *p* = .053), but not for contraceptive users (*β* = .36, *p* = .303). Similar regressions run separately for the naturally-cycling women and contraceptive users using the Wilcox estimate, Relationship Status (dichotomously coded), and the Wilcox by Relationship Status interaction term as predictor variables for the IPT pain tolerance scores revealed a significant (Wilcox x Relationship) interaction term for naturally-cycling women (*β* = −.53, *p* = .012), but not for contraceptive users (*β* = −.19, *p* = .531). These analyses show that there is an association between the probability of conception and IPT pain ratings in naturally-cycling women, and this association is moderated by the women’s current romantic relationship status.

**Figure 1:**
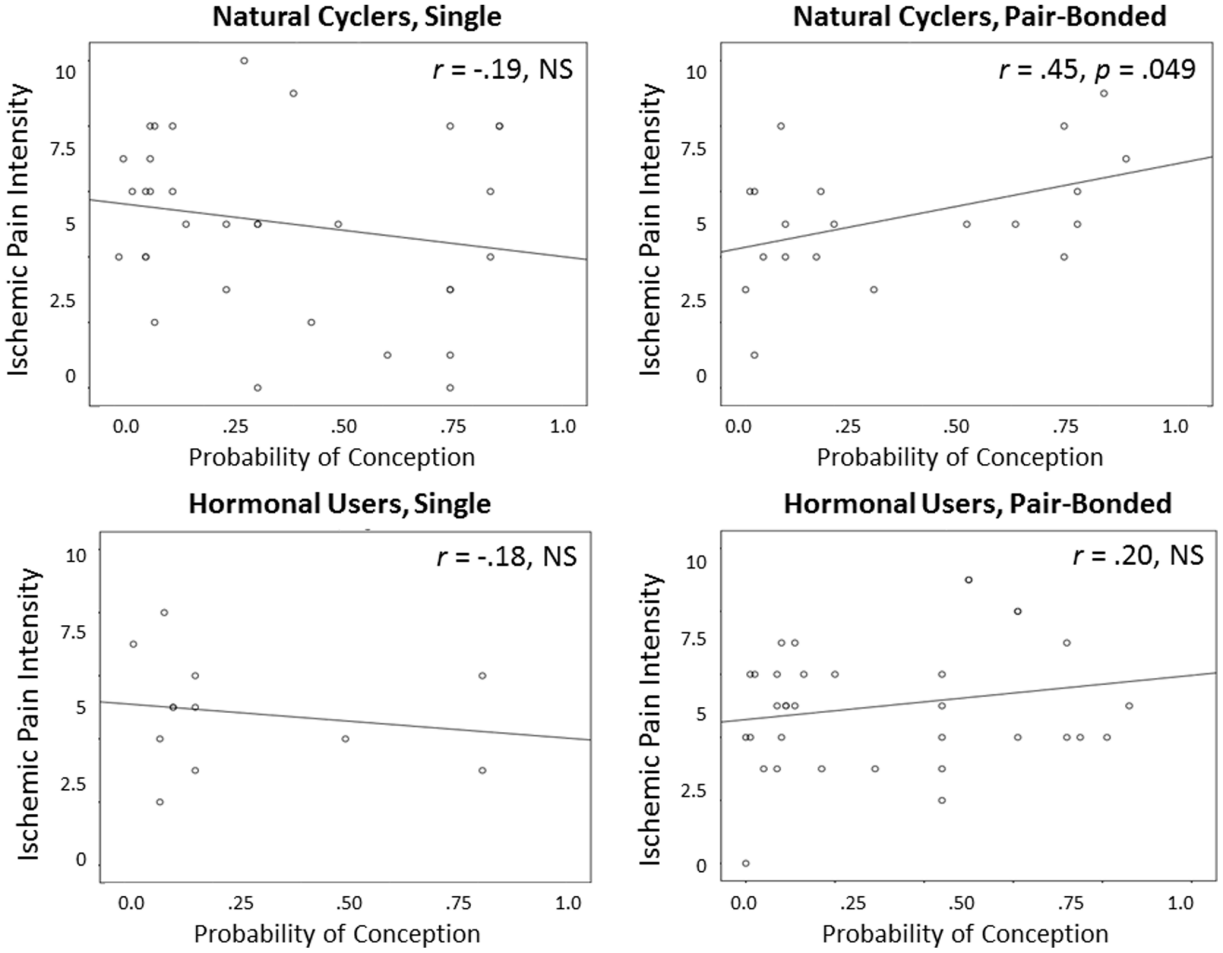
The bi-variate correlations between the probability of conception on a given calendar-day and IPT pain intensity rating for naturally cycling pair-bonded and single women and women using contraceptives. Probability of conception was calculated using the Wilcox findings which provide a precise estimate of the risk of pregnancy relative to intercourse on a given cycle day (counting from onset of previous menses; [77]).

**Figure 2:**
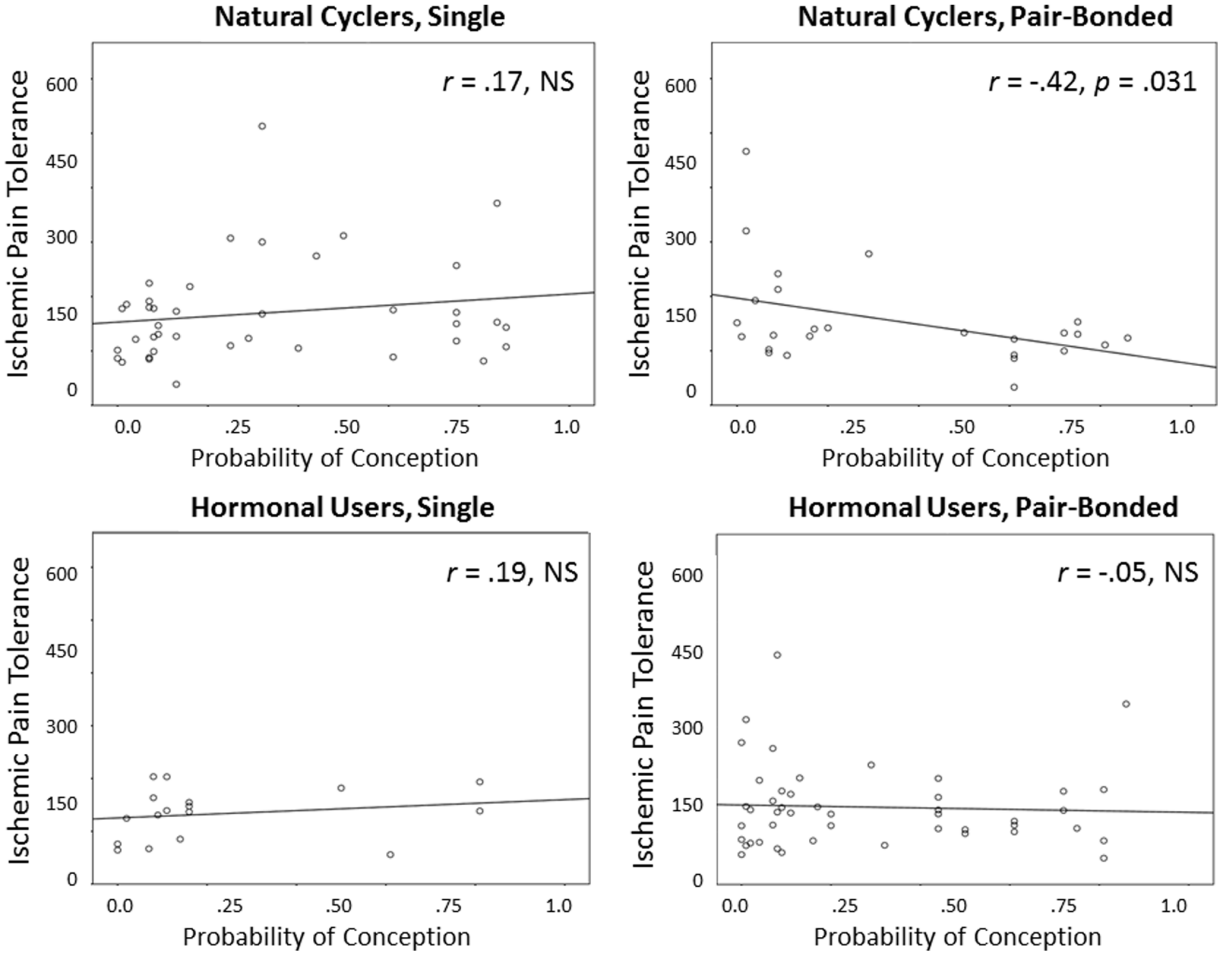
The bi-variate correlations between the probability of conception on a given calendar-day and IPT pain tolerance for naturally cycling pair-bonded and single women and women using contraceptives. Probability of conception was calculated using the Wilcox findings which provide a precise estimate of the risk of pregnancy relative to intercourse on a given cycle day (counting from onset of previous menses; [77]).

Bivariate correlations between the Wilcox estimates and the pain intensity and pain tolerance scores run separately for women who were in a romantic relationship and single women who were naturally cycling or using hormonal contraceptives are shown in Figures 1 and 2. As shown in Figure 1, there was a moderate positive correlation between the probability of conception and pain intensity for naturally-cycling, pair-bonded women (*r* = .45, *p* = .049), but not for single women or for women using contraceptives (*p*-values >.10). Similarly, Figure 2 shows a moderate negative correlation between the probability of conception and pain tolerance times for naturally cycling pair-bonded women (*r* = −.42, *p* = .031), but again not for single women or for women using contraceptives (*p*s >.10).

### Experiment 2 Results (Cold Pressor Pain Task)

Regressions run separately for the naturally-cycling women and contraceptive users using the Wilcox estimate, Relationship Status (dichotomously coded), and the Wilcox by Relationship Status interaction term as predictor variables for the mean CPT pain intensity rating, and entering CPT temperature and length of relationship as covariates, revealed a significant (Wilcox x Relationship) interaction term for the naturally-cycling women, (*β* = .71, *p* = .003), but not for contraceptive users, (*β* = <.01, *p* = .98). These analyses again show that the association between women’s probability of conception and their exogenous pain ratings is moderated by the women’s current relationship status.

**Figure 3:**
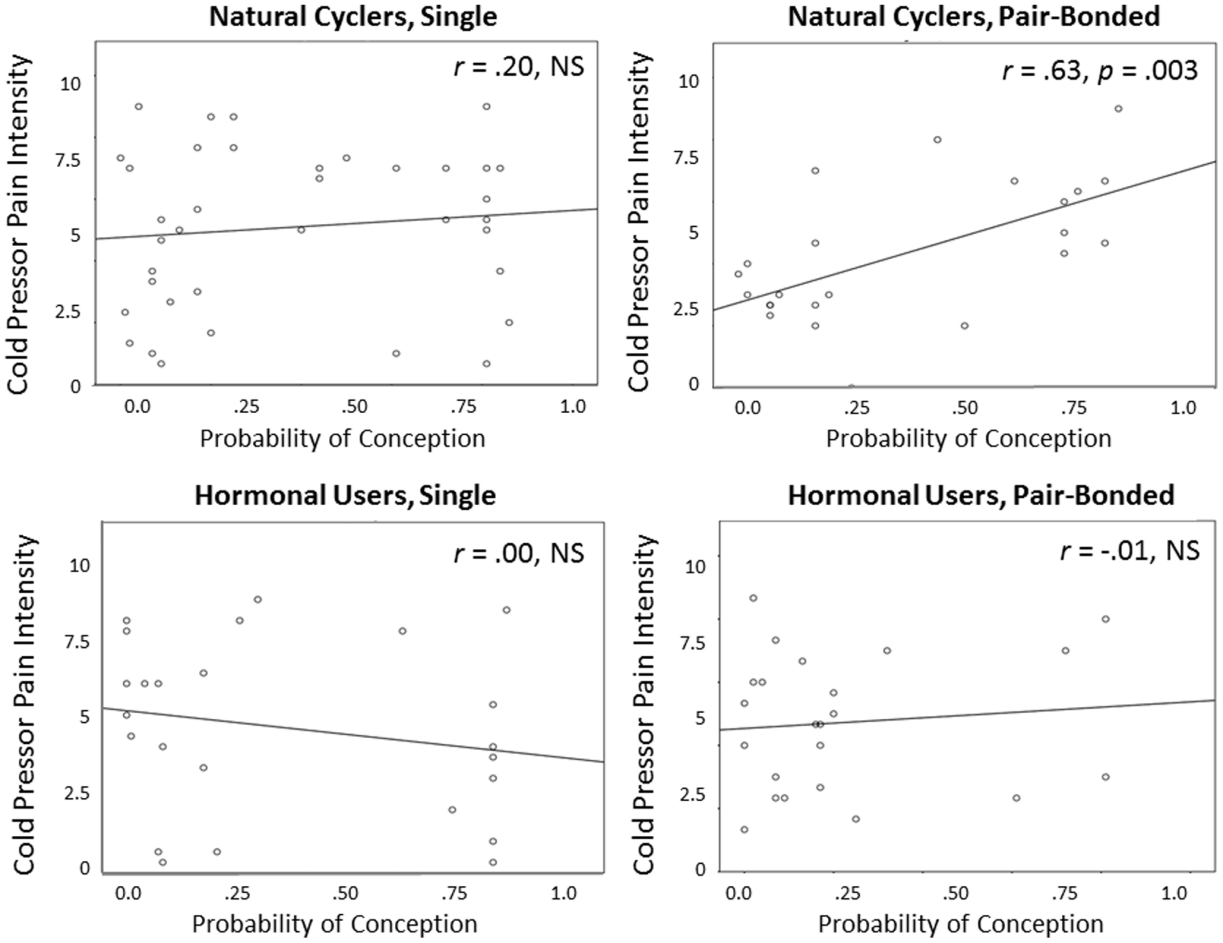
The partial correlations between the probability of conception on a given calendar-day and CPT pain intensity rating for naturally cycling pair-bonded and single women and women using contraceptives. Probability of conception was calculated using the Wilcox findings which provide a precise estimate of the risk of pregnancy relative to intercourse on a given cycle day (counting from onset of previous menses; [77]). Length of relationship was partialled out of the correlations in the pair-bonded women.

Partial correlations between the CPT pain intensity scores and the Wilcox estimates run separately for women who were in a romantic relationship and single women who were naturally cycling or using contraceptives (controlling for CPT temp and length of relationship) are shown in Figure 3. As shown, there was a moderate-to-high positive correlation between the probability of conception and pain intensity for naturally-cycling, pair-bonded women (*r* = .63, *p* = .003), but not for single women or for women using contraceptives (*p*-values >.10).

## General Discussion

These studies show for the first time that menstrual-related variability in exogenous pain sensitivities may be contingent on the woman’s current romantic relationship status. Naturally-cycling, pair-bonded women evidenced a broad pattern of dampened sensitivity to both ischemic and cold pressor pain when they had the lowest probability of conception and to experience heightened experimental pain sensitivities when they had the highest probability of conception; however, these associations did not occur for single women or women using hormonal contraceptives. The statistical magnitudes of these effects were moderate-to-large suggesting that women’s romantic relationship status may be an important factor (and basic component of women’s social network) that contributes to cyclical variability in pain sensitivity *in vitro*.

These results are consistent with the broader literature showing that cognitive percepts (e.g., reported feelings, dispositions and judgments) of external stimuli vary across the menstrual cycle in ways that would appear to facilitate selective interactions with other people. Menstrual-related changes in social cognition have been found across several domains of psychological functioning including romantic relationship preferences, relationship satisfaction, risk avoidance, and social decision-making [20], [21], [29], [33], [35], [45], [55], [57]. A social-signaling perspective of pain similarly predicts that both exogenous and endogenous pain percepts, as well as pain empathizing reactions of others heuristically operate in part at the expressive-level for advertising vulnerability and altruism attributes (respectively), and ultimately trustworthiness cues, toward intimate affiliates such as family and close friends [9], [11], [68], [69], [73]. In this sense, the occurrence of when pain that is demonstrated and responded to by others can be viewed as a symbiotic transactional process in which people interchange reciprocal demonstrations of trustworthiness cues for inducing bonding and for generally regulating relationships with trusted affiliates [12].

The current findings from two studies showed a consistent pattern for females to experience exogenous pain differently according to their relative risk of conception and depending on their current availability of a romantic relationship partner. One speculative possibility is that these patterns correspond to normative changes in endogenous pain percepts and related discomfort sensations across the menstrual cycle [73]. Since pain percepts interrupt attentional processes [14], [31], [38], humans may be limited in their ability to experience multiple, simultaneous internal versus external pain sensations at any given time [10], [31]. Thus, when women tend to naturally experience endogenous, perimenstrual discomfort (e.g., dysmenorrhea, headaches, bloating) and stress-induced negative affect during low fertility phases of the cycle [13], [19], [22], [23], [34], [44], [54], they may experience shifts in attentional resources that inhibit the ability to disengage from intrinsic discomfort percepts and diminish attentional resources available for detecting and discriminating extrinsic noxious stimuli [28], [62], [64], [73], [76]. The corresponding hypothesis is that women may tend to experience hyperalgesia (heightened pain sensation) to extrinsic noxious stimuli when they naturally experience lower levels of endogenous discomfort percepts (during high-fertility).

The next step for testing this thesis is to examine how naturally-occurring, premenstrual discomfort may be modulated, as has been demonstrated in the current study, with basic elements of women’s social environment including romantic relationship status. In a recent review of the literature on fluctuations in mood across the menstrual cycle, the authors showed mixed findings and concluded that there is no validity to the belief that women experience premenstrual mood disturbances [54]. However, no prior research has taken romantic relationship dynamics into account, which may have confounded the ability to detect such effects. From a social-signaling perspective of pain, menstrual-related fluctuations in negative affect, and possibly other cognitive and behavioral disturbances (e.g., loss of concentration, verbal fluency, balance) should be partly moderated by women’s pair-bond status and hence the availability of the types of affiliates who provide the greatest fitness incentives for expressing (uncontrollable, endogenous) pain behaviors in regular, isochronal ways that correspond with the probability of pregnancy [73].

In addition to these potentially innovative and generative implications, a discussion of study’s limitations is warranted. General methodological limitations are that: a) the cold pressor study did not control for handedness, which is known to influence CPT measurements [46]; b) reactions to the discomfort tasks might not predict reactions to other forms of painful and non-painful stimuli; c) results from American university students might not generalize to different ages, cultures, and social network structures; and d) self-reports of menstrual functioning might be biased and noisy. Finally, since the study is cross-sectional, the presumed influence of social experiences on pain sensitivity can only be considered tentative, and there are alternative hypotheses for the present findings. For example, one possibility is that the hormonal changes themselves differ between women with and without partners. Oxytocin is, on average, elevated in pair-bonded women. Oxytocin dampens pain sensitivity and people with chronic pain conditions (e.g., fibromyalgia) may have lower basal levels of oxytocin than healthy people [3], [63], [75]. Estradiol also appears to interact with oxytocin and increase its analgesic effects [2], [15], which may account for lower levels of perimenstrual-related pain during high fertility phases of the menstrual cycle. Hence, basal oxytocin or possibly other hormones (e.g., testosterone) could be a proximate mechanism that can explain each of the associations between fertility (which is associated, even if imperfectly, with estradiol) and changes in endogenous discomfort sensations in (normally ovulating) pair-bonded women. Attentional tradeoffs in the ability to experience internal versus external discomfort may then result in the corresponding and inversed pattern of associations between fertility and sensitivities to experience experimental noxious stimuli, as has been demonstrated in the present studies.

Nonetheless, the current findings have wide-reaching implications for: a) standardizing pain measurement protocols, b) understanding basic biopsychosocial pain-related processes, c) addressing clinical pain experiences in women, and d) understanding how felt pain influences, and is influenced by social relationships. There are dozens of investigations describing both positive and negligible associations between menstrual cycling and pain intensity, and the current study highlighted a significant individual-level confound, pair-bonding status, which has not been controlled in most previous studies. Moreover, pain-specific brain activity has been shown to vary across the menstrual cycle [60], [65], and a better understanding of the role of social psychological processes may be important for this and similar lines of basic research. The current study also has implications for better understanding the epidemiology of why women have quantitatively and qualitatively distinct pain experiences than men, including a variety of discomfort sensations that are associated with menstruation (e.g., dysmenorrhea, headaches, bloating [23], [67]). Finally, the current results may warrant further research on how changes in somatic (e.g., sensory and perceptual) functioning, including exogenous and endogenous pain sensitivities which can occur in both the presence and absence of physical tissue-damage, may share reciprocal relationships with the individual’s social experiences. It is possible, for instance, that humans evolved differential sensitivities to experience distinct types of pain percepts in coordination with social cohesion, dissolution, and reconciliation experiences, perhaps operating differently in males and females, at different stages of the human lifespan.

## References

[1] Adler G, Gattaz WF (1993) Pain perception threshold in major depression. Biol Psychiatry 34: 687–9. 8292672 10.1016/0006-3223(93)90041-b

[2] Amico JA, Seif SM, Robinson AG (1981) Oxytocin in human plasma: Correlation with neurophysin and stimulation with estrogen. J Clin Endocrinol Metab 52: 988–993. 7228998 10.1210/jcem-52-5-988

[3] Anderberg UM, Uvnäs-Moberg K (2000) Plasma oxytocin levels in female fibromyalgia syndrome patients. Z Rheumatolog 59: 373–379. 10.1007/s003930070045 11201002

[4] Bartley EJ, Rhudy JL (2013) Comparing pain sensitivity and the nociceptive flexion reflex threshold across the mid-follicular and late-luteal menstrual phases in healthy women. Clin J Pain 29: 154–161. 22688607 10.1097/AJP.0b013e31824c5edb

[5] Baeyer CL, Piira T, Chambers CT, Trapanotto M, Zeltzer LK (2005) Guidelines for the cold pressor task as an experimental pain stimulus for use with children. J Pain 6: 218–227. 15820909 10.1016/j.jpain.2005.01.349

[6] Block AR, Kremer EF, Gaylor M (1980) Behavioral treatment of chronic pain: The spouse as a discriminative cue for pain behavior. Pain 9: 243–252. 7454386 10.1016/0304-3959(80)90011-1

[7] Bressan P, Stranieri D (2008) The best men are (not always) already taken: female preference for single versus attached males depends on conception risk. Psychol Sci 19: 145–151. 18271862 10.1111/j.1467-9280.2008.02060.x

[8] Cano A, Barterian JA, Heller JB (2008) Empathic and nonempathic interaction in chronic pain couples. Clin J Pain 24: 678–684. 18806532 10.1097/AJP.0b013e31816753d8 PMC2562912

[9] Cano A, Williams ACD (2010) Social interaction in pain: Reinforcing pain behaviors or building intimacy? Pain 149: 9–11. 19892466 10.1016/j.pain.2009.10.010 PMC2834842

[10] Chan SCC, Chan CCH, Kwan ASK, Ting KH (2012) Orienting attention modulates pain perception: An ERP study. PLoS One 7: e40215. 22768257 10.1371/journal.pone.0040215 PMC3387012

[11] Craig KD (2009) The social communication model of pain. Canadian Psychology/Psychologie Canadienne 50: 22–32.

[12] Craig KD, Versloot J, Goubert L, Vervoort T, Crombez G (2010) Perceiving pain in others: Automatic and controlled mechanisms. J Pain 11: 101–108. 19962352 10.1016/j.jpain.2009.08.008

[13] Davydov DM, Shapiro D, Goldstein IB, Chicz-DeMet A (2007) Moods in everyday situations: effects of combinations of different arousal-related factors. J Psychosom Res 62: 321–329. 17324683 10.1016/j.jpsychores.2006.10.021

[14] Eccleston C, Crombez G (1999) Pain demands attention: a cognitive-affective model on the interruptive function of pain. Psychol Bull 125: 356–66. 10349356 10.1037/0033-2909.125.3.356

[15] Evans JJ, Reid RA, Wakeman SA, Croft LB, Benny PS (2003) Evidence that oxytocin is a physiological component of LH regulation in non-pregnant women. Human Reproduction 18: 1428–1431. 12832367 10.1093/humrep/deg291

[16] Fillingim RB, Doleys DM, Edwards RR, Lowery D (2003) Spousal responses are differentially associated with clinical variables in women and men with chronic pain. Clin J Pain 19: 217–224. 12840615 10.1097/00002508-200307000-00004

[17] Fillingim RB, King CD, Ribeiro-Dasilva MC, Rahim-Williams B, Riley III JL (2009) Sex, gender, and pain: a review of recent clinical and experimental findings. Pain 10: 447–485. 10.1016/j.jpain.2008.12.001 PMC2677686 19411059

[18] Flor H, Kerns RD, Turk DC (1987) The role of spouse reinforcement, perceived pain, and activity levels of chronic pain patients. J Psychosom Res 31: 251–259. 3585827 10.1016/0022-3999(87)90082-1

[19] French L (2005) Dysmenorrhea. American Family Physician 71: 285–291. 15686299

[20] Gangestad SW, Thornhill R (1998) Menstrual cycle variation in women’s preferences for the scent of symmetrical men. Proc R Soc Lond B 265: 927–933. 10.1098/rspb.1998.0380 PMC1689051 9633114

[21] Garver-Apgar CE, Gangestad SW, Thornhill R (2008) Hormonal correlates of women’s mid-cycle preference for the scent of symmetry. Evol Human Behav 29: 223–232.

[22] Gingnell M, Morell A, Bannbers E, Wikstrom J, Sundström, et al (2012) Menstrual cycle effects on amygdala reactivity to emotional stimulation in premenstrual dysphoric disorder. Hormones Behavior 62: 400–406. 22814368 10.1016/j.yhbeh.2012.07.005

[23] Iacovides S, Baker FC, Avidon I, Bentley A (2013) Women with dysmenorrhea are hypersensitive to experimental deep muscle pain across the menstrual cycle. J Pain 13: 973–975. 10.1016/j.jpain.2013.04.010 23769507

[24] Kállai I, Barke A, Voss U (2004) The effects of experimenter characteristics on pain reports in women and men. Pain 112: 142–147. 15494194 10.1016/j.pain.2004.08.008

[25] Kerns RD, Rosenberg R, Otis JD (2002) Self-appraised problem solving and pain-relevant social support as predictors of the experience of chronic pain. Ann Behav Med 24: 100–105. 12054314 10.1207/S15324796ABM2402_06

[26] Kowalczyk WJ, Sullivan MA, Evans SM, Bisaga AM, Vosburg SK, et al (2012) Sex differences and hormonal influences on response to mechanical pressure pain in humans. J Pain 11: 330–342. 10.1016/j.jpain.2009.08.004 PMC6174694 19853526

[27] Kuba T, Quinones-Jenab V (2005) The role of female gonadal hormones in behavioral sex differences in persistent and chronic pain: clinical versus preclinical studies. Brain Res Bull 66: 179–88. 16023915 10.1016/j.brainresbull.2005.05.009

[28] Lariviere C, Butler H, Sullivan MJL, Fung J (2013) An exploratory study on the effect of pain interference and attentional interference on neuromuscular responses during rapid arm flexion movements. Clin J Pain 29: 265–275. 23369930 10.1097/AJP.0b013e318250ed6f

[29] Larson CM, Haselton MG, Gildersleeve KA, Pillsworth EG (2013) Changes in women’s feelings about their romantic relationships across the ovulatory cycle. Hormones Behav 63: 128–135. 10.1016/j.yhbeh.2012.10.005 23085495

[30] Lautenbacher S, Spernal J, Schreiber W, Krieg JC (1999) Relationship between clinical pain complaints and pain sensitivity in patients with depression and panic disorder. Psychosom Med 61: 822–827. 10593634 10.1097/00006842-199911000-00015

[31] Legrain V, Van Damme S, Eccleston C, Davis KD, Seminowicz DA, et al (2009) A neurocognitive model of attention to pain: Behavioral and neuroimaging evidence. Pain 144: 230–232. 19376654 10.1016/j.pain.2009.03.020

[32] Levine FM, De Simone LL (1991) The effects of experimenter gender on pain report in male and female subjects. Pain 44: 69–72. 2038491 10.1016/0304-3959(91)90149-R

[33] Lukaszewski AW, Roney JR (2009) Estimated hormones predict women’s mate preferences for dominant personality traits. Person Individual Diff 47: 191–196.

[34] MacGregor EA, Hackshaw A (2004) Prevalence of migraine on each day of the natural menstrual cycle. Neurology 63: 351–353. 15277635 10.1212/01.wnl.0000133134.68143.2e

[35] Macrae CN, Alnwick KA, Milne AB, Schloerscheidt AM (2002) Person perception across the menstrual cycle: Hor-monal influences on social-cognitive functioning. Psychol Sci 13: 532–536. 12430837 10.1111/1467-9280.00493

[36] McClelland LE, McCubbin JA (2008) Social influence and pain response in women and men. J Behav Med 31: 413–420. 18587638 10.1007/s10865-008-9163-6

[37] Mitchell LA, MacDonald RAR, Brodie EE (2004) Temperature and the cold pressor test. J Pain 5: 231–238. 10.1016/j.jpain.2004.03.004 15162346

[38] Moseley GL, Nicholas MK, Hodges PW (2004) Pain differs from non-painful attention-demanding or stressful tasks in its effect on postural control patterns of trunk muscles. Exp Brain Res 156: 64–71. 14689133 10.1007/s00221-003-1766-0

[39] Newton-John TRO (2002) Solicitousness and chronic pain: A critical review. Pain Rev 9: 7–27.

[40] Norbeck JS, Lindsey AM, Carrieri VL (1981a) The development of an instrument to measure social support. Nursing Res 30: 264–269. 7027185

[41] Norbeck JS, Lindsey AM, Carrieri VL (1981b) Further development of The Norbeck social support questionnaire: normative data and validity testing. Nursing Res 32: 4–9. 6549842

[42] Okifuji A, Turk DC (2006) Sex hormones and pain in regularly menstruating women with fibromylgia syndrome. J Pain 7: 851–859. 17074627 10.1016/j.jpain.2006.04.005

[43] Orbach-Zinger S, Ginosar Y, Sverdlik J, Treitel C, MacKersey K, et al (2012) Partner’s presence during initiation of epidural labor analgesia does not decrease maternal stress: a prospective randomized controlled trial. Anesth Analg 114: 654–60. 22253271 10.1213/ANE.0b013e318241f4f3

[44] Ossewaarde L, van Wingen GA, Rijpkema M, Backstrom T, Hermans EJ, et al. 2013 Menstrual cycle-related changes in amygdala morphology are associated with changes in stress sensitivity. Hum Brain Mapping 34: 1187–1193. 10.1002/hbm.21502 PMC6870478 22162177

[45] Pawlowski B, Jasienska G (2005) Women’s preferences for sexual dimorphism in height depend on menstrual cycle phase and expected duration of relationship. Biol Psychol 70: 38–43. 16038772 10.1016/j.biopsycho.2005.02.002

[46] Pud D, Golan Y, Pesta R (2009) Hand dominancy- A feature affecting sensitivity to pain. Neurosci Let 467: 237–240. 19853018 10.1016/j.neulet.2009.10.048

[47] Radloff LS (1997) The CES-D scale: A self-report depression scale for research in the general population. Applied Psych Measure 1: 385–401.

[48] Rezaii T, Hirschberg AL, Carlström K, Ernberg M (2012) The influence of menstrual phases on pain modulation in healthy women. J Pain 13: 646–655. 22634142 10.1016/j.jpain.2012.04.002

[49] Riley III JL, Robinson ME, Wise EA, Myers CD, Fillingim RB (1998) Sex differences in the perception of noxious experimental stimuli: A meta-analysis. Pain 74: 181–187. 9520232 10.1016/s0304-3959(97)00199-1

[50] Ring C, Veldhuijzen van Zanten J, Kavussanu M (2009) Effects of sex, phase of the menstrual cycle and gonadal hormones on pain in healthy humans. Biol Psychol 8: 189–191. 10.1016/j.biopsycho.2009.04.004 19393713

[51] Romano JM, Jensen MP, Schmaling KB, Hops H, Buchwald DS (2009) Illness behaviors in patients with unexplained chronic fatigue are associate with significant other responses. Journal of Behavioral Medicine 32: 558–569. 19915971 10.1007/s10865-009-9234-3

[52] Romano JM, Jensen MP, Turner JA, Good AB, Hops H (2000) Chronic pain patient-partner interactions: Further support for a behavioral model of chronic pain. Behavior Therapy 31: 415–440.

[53] Romano JM, Turner JA, Jensen MP, Friedman LS, Bulcroft RA, et al (1995) Chronic pain patient-spouse behavioral interactions predict patient disability. Pain 63: 353–360. 8719536 10.1016/0304-3959(95)00062-3

[54] Romans S, Clarkson R, Einstein G, Petrovic M, Stewart D (2012) Mood and the menstrual cycle: a review of prospective data studies. Gend Med 9: 361–384. 23036262 10.1016/j.genm.2012.07.003

[55] Roney JR, Simmons ZL (2008) Women’s estradiol predicts preference for facial cues of men’s testosterone. Hormon Behav 53: 14–19. 10.1016/j.yhbeh.2007.09.008 17950291

[56] Ruau D, Liu LY, Clark D, Angst MS, Butte AJ (2011) Sex differences in reported pain across 11,000 patients captured in electronic medical records. J Pain 13: 228–234. 10.1016/j.jpain.2011.11.002 PMC3293998 22245360

[57] Senior C, Lau A, Butler MR (2007) The effects of the menstrual cycle on social decision making. International J Psychophysiology 63: 186–191. 10.1016/j.ijpsycho.2006.03.009 16815580

[58] Stening K, Eriksson O, Wahren L, Berg G, Hammar M, et al (2007) Pain sensations to the cold pressor test in normally menstruating women: comparison with men and relation to menstrual phase and serum sex steroid levels. American J Physiology. Regulatory Integrative and Comparative Physiology 293: 1711–1716. 10.1152/ajpregu.00127.2007 17652363

[59] Teepker MM, Peters MM, Vedder HH, Schepelmann KK, Lautenbacher SS (2010) Menstrual variation in experimental pain: Correlation with gonadal hormones. Neuropsychobiology 61: 131–140. 20110738 10.1159/000279303

[60] de Tommaso M, Valeriani M, Sardaro M, Serpino C, Fruscolo OD, et al (2009) Pain perception and laser evoked potentials during menstrual cycle in migraine. J Headache Pain. 10: 423–429. 10.1007/s10194-009-0150-2 PMC3476220 19763770

[61] Tousignant-Laflamme Y, Marchand S (2009) Excitatory and inhibitory pain mechanisms during the menstrual cycle in healthy women. Pain 146: 47–55. 19592167 10.1016/j.pain.2009.06.018

[62] Tracey I, Mantyh PW (2007) The cerebral signature for pain perception and its modulation. Neuron 55: 377–391. 17678852 10.1016/j.neuron.2007.07.012

[63] Uryvaev YV, Petrov GA (1996) Extremely low doses of oytocin reduce pain sensitivity in men. Bull Experimental Biol Med 122: 1071–1073.

[64] Van Damme S, Crombez G, Eccleston C (2004) The anticipation of pain modulates spatial attention: evidence for pain-specificity in high-pain catastrophizers. Pain 111: 392–399. 15363884 10.1016/j.pain.2004.07.022

[65] Veldhuijzen DS, Keaser ML, Traub DS, Zhuo J, Gullapalli RP, et al (2013) The role of circulating sex hormones in menstrual cycle-dependent modulation of pain-related brain activation. Pain 154: 548–59. 23528204 10.1016/j.pain.2012.12.019 PMC3608932

[66] Vervoort T, Goubert L, Eccleston C, Verhoeven K, De Clercq A, et al (2008) The effects of parental presence upon the facial expression of pain: the moderating role of child pain catastrophizing. Pain 138: 277–85. 18243557 10.1016/j.pain.2007.12.013

[67] Vetvik KG, MacGregor EA, Lundqvist C, Russell MB (2010) Self-reported menstrual migraine in the general population. J Headache Pain 11: 87–92. 20186561 10.1007/s10194-010-0197-0 PMC3452281

[68] Vigil JM (2009a) The socio-relational framework of expressive behaviors as an integrative psychological paradigm. Behav Brain Sci 32: 408–428. 10.1017/S0140525X09991075 19825246

[69] Vigil JM (200b) A socio-relational framework of sex differences in the expression of emotion. Behav Brain Sci 32: 375–390. 19825246 10.1017/S0140525X09991075

[70] Vigil JM, Coulombe P (2011) Biological sex and social setting affects pain intensity and observational coding of other people’s pain behaviors. Pain 152: 2125–2130. 21664763 10.1016/j.pain.2011.05.019

[71] Vigil JM, Rowell LN, Alcock J, Maestes R (in press) Laboratory personnel gender and cold pressor apparatus affect subjective pain reports. Pain Res Manag. 10.1155/2014/213950 PMC3938346 24367796

[72] Vigil JM, Rowell N, Chouteau S, Chavez A, Jaramillo E, et al (2013) Sex differences in how social networks and relationship quality influence experimental pain sensitivity. PLOS ONE 8(11): e78663 doi:10.1371/journal.pone.0078663 24223836 10.1371/journal.pone.0078663 PMC3818490

[73] Vigil JM, Strenth C (in press) No pain, no social gains: A social-signaling perspective of human pain behaviors. World J Anesthesiology.

[74] von Baeyer CL, Piira T, Chambers CT, Trapanotto M, Zeltzer LK (2005) Guidelines for the cold pressor task as an experimental pain stimulus for use with children. J Pain 6: 218–227. 15820909 10.1016/j.jpain.2005.01.349

[75] Wang Y, Yuan Y, Yang J, Wang C, Pan Y, et al (2013) The interaction between the oxytocin and pain modulation in headache patients. Neuropeptides 47: 93–97. 23375440 10.1016/j.npep.2012.12.003

[76] Wiech K, Seymour B, Kalisch R, Enno SK, Koltzenburg M, et al (2005) Modulation of pain processing in hyperalgesia by cognitive demand. Neuroimage 27: 59–69. 15978845 10.1016/j.neuroimage.2005.03.044

[77] Wilcox AJ, Dunson DB, Weinberg CR, Trussell J, Baird DD (2001) Likelihood of conception with a single act of intercourse: providing benchmark rates for assessment of post-coital contraceptives. Contraception 63: 211–215. 11376648 10.1016/s0010-7824(01)00191-3

